# The Ticking Clock of Aortic Root Replacement - Single-Center Experience After Urgent and Emergent Aortic Root Replacement Using the BioIntegral and Freestyle™ Bioconduits

**DOI:** 10.21470/1678-9741-2024-0307

**Published:** 2025-05-23

**Authors:** Konstantina Spetsotaki, Jingjing Shi, Ajay Moza, Matthias Menne, Ali Aljalloud

**Affiliations:** 1 Department of Thoracic Surgery, University Hospital Zurich, Raemistrasse, Zurich, Switzerland; 2 Department of Cardiovascular Surgery, RWTH University Hospital Aachen, Aachen, Germany; 3 Department of Cardiovascular Engineering, Institute of Applied Medical Engineering, Medical Faculty, RWTH Aachen University, Aachen, Germany; 4 Department of Cardiology, Nephrology, and Internal Intensive Care, Rhein-Maas Hospital, Würselen, Germany

**Keywords:** Thoracic Aorta, Cardiopulmonary Bypass, Aortic Valve, Bioprosthesis, Hospitals, Outpatients

## Abstract

**Introduction:**

Aortic root pathologies needing full aortic root replacement are challenging entities correlated to high morbidity and mortality due to their complexity and mostly refer to high-risk patients. In this retrospective study, we report our surgical experience and clinical results of patients undergoing a Bentall procedure as primary or reoperative surgery with the application of aortic bioconduits.

**Methods:**

Patients who underwent full aortic root replacement utilizing either BioIntegral (BI) or Medtronic Freestyle™ (FS) bioconduit in the Cardiothoracic Surgery Department of the University Hospital Aachen RWTH from January 2015 until September 2020, in an urgent or emergency setting, were analyzed and followed up until December 2023.

**Results:**

Twenty-six patients underwent aortic root replacement with bioconduits (N=11 with BI, N=15 with FS) in our center. Twenty-three cases were of infective cause, and three were of noninfective cause; 30.76% were urgent, and 69.23% were emergency cases. Two (7.70%) patients died during operation due to irreversible aortic root damage. In-hospital and 30-day mortality rates were four out 26 (15.4%) patients. The mean follow-up time for all the patients was 52.01 ± 39.41 months. Patients who received a primary aortic root replacement had significantly higher survival than redo cases. BI surgery needed longer cardiopulmonary bypass times.

**Conclusion:**

Clinical outcome was equal for both bioconduits. Further studies with larger cohorts are needed for deeper insights into this complex entity.

## INTRODUCTION

**Table:** 

Abbreviations, Acronyms & Symbols				
AAR	= Ascending aorta replacement		IE	= Infective endocarditis
AKI	= Acute kidney injury		LV	= Left ventricular
AMC	= Aortomitral curtain		LSB	= Left septal block
AMCR	= Aortomitral curtain reconstruction		MPG	= Mean pressure gradient
AR	= Aortic root		MSSA	= Methicillin-susceptible *Staphylococcus aureus*
ARA	= Aortic root abscess		MVR	= Mitral valve repair
ARR	= Aortic root replacement		MVRp	= Mitral valve replacement
AV	= Aortic valve		PAOD	= Peripheral arterial occlusive disease
AVR	= Aortic valve replacement		POD	= Postoperative day
BI	= BioIntegra		PPG	= Peak pressure gradient
BMI	= Body mass index		RCA	= Right coronary artery
BSA	= Body surface area		ROSC	= Return of spontaneous circulation
CABG	= Coronary artery bypass grafting		SCAAR	= Supracoronary ascending aorta replacement
CPB	= Cardiopulmonary bypass		STS	= Society of Thoracic Surgeons
EF	= Ejection fraction		TAPSE	= Tricuspid annular plane systolic excursion
EuroSCORE II	= European System for Cardiac Operative Risk Evaluation		TAVI	= Transcatheter aortic valve implantation
FS	= Freestyle™		TVR	= Tricuspid valve reconstruction
HLM	= Heart-lung machine		VT	= Ventricular tachycardia
ICU	= Intensive care unit			

Aortic root (AR) and aortic valve (AV) pathologies encompass a wide spectrum including noninfective and infective diseases, such as AR dilation, dissection, and infective endocarditis (IE) which constitute a surgical challenge, with the indication for AR replacement (ARR)^[1^,^2]^. De Bono and Bentall first described composite valve and graft replacement in 1968^[3]^.

Patient age is one of the strongest deciding factors for the type of prosthesis. The guidelines set forth by the European Society of Cardiology designate 65 years as the threshold for initial consideration when selecting biological AV prostheses^[4^,^5]^. The field of development and evolution of aortic prostheses has seen great progress, with the stentless bioprostheses having superior hemodynamics and beneficial left ventricular remodeling results^[6^,^7]^. However, the results from previous studies regarding AV prostheses should not be generalized for patients needing ARR due to the differences between blood flow and pathophysiological changes adaptation after ARR^[8]^.

Bioprosthetic valves can degenerate structurally, requiring a full redo ARR. Older patients are requiring more redo cardiac operations, leading to an increase in AR reoperations^[9]^. As transcatheter aortic valve implantation (TAVI) rates continue to rise in the modern era of transcatheter cardiac surgery, it is expected that a greater number of young patients will receive xenografts. The utilization of Bioroots is highly rewarded in cases requiring redo surgeries, as it facilitates the application of transcatheter alternatives over an extended period^[10^-^12]^. Moreover, given the benefits of avoidance of anticoagulation and the alternative of valve-in-valve TAVI instead of a high-risk reoperation, the use of Bioroots technology is becoming more prevalent as a popular solution for patients in need of ARR^[8]^. And although first generation xenografts had a limited durability^[8]^, new generation bioconduits have proven to be equivalent to both their mechanical and stented conduits analogs, in terms of long-term outcome, reoperation rates, functional performance, and longevity^[13]^.

However, the commercially available bioconduits that can be utilized for the Bentall procedure are still limited^[14^,^15]^. One of the most extensively studied and frequently utilized commercially available bioconduits is the Freestyle™ (FS) conduit by Medtronic (Irvine, California, United States of America), offering a xenogeneic whole ARR biological conduit^[12^,^16]^. An alternative is by surgeons' choice, the application of self-manufactured conduits that were created by suturing biological aortic prostheses onto a Dacron® graft^[17]^. Another commonly utilized complete biologic substitute was the BioIntegral (BI) prosthesis developed by BioIntegral Surgical, Inc. (Mississauga, Ontario, Canada). The BI is an enhanced iteration of the Shelhigh® bioconduit developed by Shelhigh®, Inc. (Milburn, New Jersey, United States of America). It is composed of a compound xenograft made up of a porcine AV and bovine pericardium, which has been treated with No-React® preservation technology, providing reduced rates of reinfection and improved biocompatibility^[18]^.

AR IE carries an ominous prognosis and recurrence rates^[19]^. Moreover, reoperative ARR conducted in a number of complex clinical scenarios is related to high morbidity and mortality^[20]^. There is still limited evidence regarding the clinical results. More data is needed comparing the results after the application of different bioconduits in challenging urgent or emergency cases of ARR. Here, we report our experience and the clinical results of patients undergoing urgent or emergent complete ARR surgery with BI and FS bioconduits.

## METHODS

### Data and Patient Cohort

We conducted a retrospective collection of patient data, including demographic details, clinical outcomes, perioperative protocols, postoperative recovery, imaging findings, and laboratory results from our institutional databank. The research was granted approval by the Ethics Committee of RWTH University Hospital Aachen, in compliance with the IRBP regulations 10/2014 and EK151/09-Version-1.3 guidelines. Due to the retrospective design of our study, the requirement for informed consent was waived by our institutional review board. In this single-center, retrospective, observational study, adult participants (aged over 18 years) who underwent an isolated or combined full ARR with a bioconduit of either FS or BI type, in our Department of Cardiothoracic Surgery, University Hospital of Aachen RWTH, from January 2015 until September 2020, were analyzed and followed up until December 2023.

We obtained subsequent data by conducting direct telephone interviews with either the referring cardiologists and general physicians or the patients. All patients who survived underwent transthoracic assessment using two-dimensional echocardiography in accordance with the institutional protocol post-discharge, at six months post-surgery, and subsequently on an annual basis.

### Statistical Analysis

All quantitative variables were presented by mean values and standard deviations, and all qualitative variables were presented as frequencies and percentages (N, %). Comparisons between continuous variables of two groups were conducted using the Student’s *t*-test analysis for the comparison of categorical variables; Chi-square (X^^2^^) test was used. Kaplan-Meier survival analysis was conducted for different cohorts throughout the duration of the follow-up period. *P* values < 0.05 were considered as statistically significant. IBM Corp Released 2023, IBM SPSS Statistics for Windows, version 29.0, Armonk, NY: IBM Corp. was used for all the analyses.

### Surgical Technique

The same experienced team of cardiac surgeons performed all procedures. The team decided on the surgery technique after daily meeting evaluation. The selection of the bioconduit was contingent upon the surgeon's individual preference. Standard surgical strategies with median sternotomy approach and application of cardiopulmonary bypass (CPB) were used. The selection of the cannulation technique for CPB was dictated by the patient's medical condition and the complexity of the surgical procedure, with consideration given to direct aortic, subclavian, or femoral cannulation. When needed, in redo cases, the CPB was applied before resternotomy through peripheral cannulation. Depending on the extent of the anticipated aortic reconstruction required, the optimal cannulation and cerebral protection strategy were implemented in each case. Mild (28-32 °C) or moderate (25-28 °C) hypothermia were implemented. During hemiarch or total arch replacement procedures, hypothermic circulatory arrest with antegrade cerebral perfusion was employed. Myocardial preservation and cardiac arrest were achieved with the use of antegrade and optional retrograde crystalloid cardioplegia (Custodiol®, Dr. F. Köhler Chemie, Bensheim, Germany). The aorta was incised 4 cm above the right coronary ostium, and the AR was extensively freed from the surrounding tissue, down to the subvalvular plane. The prosthetic or native AV was completely excised, and both coronary ostia were prepared, suspended, and mobilized as buttons. The excision of the aortic sinuses was followed by the selection of an appropriate biological pericardial conduit. The BI conduit was implanted with single, interrupted 3-0 Ethibond® (Ethicon, Johnson & Johnson Medical, Brussels, Belgium) pledged U-stitch sutures. Everted sutures with pledgets located outside of the conduit were used. For the FS application, running 3-0 PROLENE® (Ethicon) was applied. On the former left ostium of the FS graft, the left coronary artery button was implanted. In cases of BI, the holes for coronary buttons were cut open on the respective sinuses of the conduit. Great care was taken to prevent any tension or kinks on the right button. A 4 mm punch was used for the opening pericardial conduit, matching the left coronary button. After trimming the coronary button, a 2-3 mm aortic cuff was ensured, followed by a parachute anastomosis with a 5-0 (or 6-0) polypropylene running technique.

In the case of AR abscess (ARA), the proximal suture line utilized for the implantation of the bioconduit must run continuously in the left ventricular outflow tract along the robust nadir of the abscess. The bites taken during this procedure encompass both the complete muscular septum and a small margin of AML. In some cases, a pericardial plastic patch of the aortomitral curtain (AMC) was applied. The right coronary artery was re-attached, and the bioconduit was shortened to match the aorta, then sutured with 4-0 polypropylene. Once all distal anastomoses were finished, the patient was gradually rewarmed.

Valve size was determined through imaging and confirmed during surgery. Priority is given to maximizing orifice area and optimizing the hemodynamics in aneurysmal root disease cases.

## RESULTS

Twenty-six patients are reported in this study; 23 (88.46%) patients had an infective underlying cause; 21 (80.8%) were males, and five (19.2%) were females. Demographic and clinical data are detailed and presented in Table 1. The average age was 62.31 ± 16.61 years in the total cohort, 58.13 ± 15.82 in the FS group, and 68.00 ± 16.66 in the BI group; 30.76% were urgent and 69.23% were emergency cases.

**Table 1:** Baseline characteristics.

Parameters	BI (N=11)	FS (N=15)	*P*-value
Age at time of current surgery	68.00 ± 16.66	58.13 ± 15.82	0.138
Male sex	9 (81.8%)	12 (80%)	0.907
Height, m	172.91 ± 7.61	175.20 ± 6.61	0.421
Weight, kg	78.00 ± 12.75	78.67 ± 9.47	0.879
BMI, kg/m^2^	26.16 ± 4.85	25.61 ± 2.80	0.718
BSA, m^2^	1.90 ± 0.18	1.94 ± 0.13	0.479
PAOD	1 (9.1%)	2 (13.3%)	0.738
EuroSCORE II	23.76 ± 24.40	11.53 ± 10.91	0.097
STS score	4.84 ± 6.25	1.58 ± 0.71	0.048
Previous cardiac surgery	7 (63.6%)	9 (60%)	0.851
Previous aortic surgery			0.343
Previous Bentall procedure	1 (9.1%)	2 (13.3%)	
Previous David procedure with hemiarch replacement	0 (0%)	1 (5.7%)	
Previous David procedure	1 (9.1%)	0 (0%)	
Previous SCAAR by type A dissection	1 (9.1%)	0 (0%)	
Aortic valve			0.908
Native	5 (45.4%)	5 (33.3%)	
Previous AV prosthesis	4 (36.4%)	7 (46.7%)	
Previous AR conduit	1 (9.1%)	2 (13.3%)	
Previous David procedure, preserved AV	1 (9.1%)	1 (6.6%)	
ARA presence			0.599
ARA	7 (63.6%)	8 (53.3%)	
Non-ARA	4 (36.4%)	7 (46.7%)	
Redo surgery (year after last cardiac surgery)	4.33 ± 6.92	2.13 ± 2.07	0.329
Embolic complications before surgery			0.325
Νone	11 (100%)	11 (73.3%)	
Pulmonary septic embolic complications due to IE	0 (0%)	2 (13.3%)	
Kidney, spleen, and cerebral septic embolic complication due to IE	0 (0%)	1 (6.7%)	
Limb and intestinal ischemia due to septic cardiac embolization	0 (0%)	1 (6.7%)	
Main diagnosis			0.944
Native AR IE + ARA	2(18.2%)	2 (13.3%)	
Native AV IE without ARA	2 (18.2%)	1 (6.7%)	
AV prosthesis IE after AVR + ARA	3 (27.3%)	4 (%)	
AV prosthesis IE after AVR without ARA	1 (9.1%)	3 (%)	
IE after previous David procedure + ARA	1 (9.1%)	1 (6.7%)	
Conduit IE after Bentall surgery without ARA	0 (0.0%)	1(6.7%)	
Conduit IE after Bentall surgery + ARA	1 (9.1%)	1(6.7%)	
Aorta ascending aneurysm with mixed severe AV disease, noninfective	1 (9.1%)	2 (13.3%)	
Underlying condition			0.364
Infective	9 (81.8%)	14 (93.3%)	
Noninfective	2 (18.2%)	1 (6.7%)	
Urgency status			0.597
Urgent	4 (36.4%)	4 (26.7%)	
Emergency	7 (63.6%)	11 (73.3%)	
Blood culture			0.139
Positive	3 (37.3%)	10 (66.7%)	
Negative	3 (27.3%)	2 (13.3%)	
IE focus			0.395
Unknown	6 (54.5%)	10 (66.7%)	
Pacemaker IE	0 (0%)	1 (6.7%)	
Orthopedic implant focus	0 (0%)	1 (6.7%)	
Thrombophlebitis	0 (0%)	1 (6.7%)	
Spondylodiscitis	1 (9.1%)	0 (0%)	
Pathogens			0.215
Not found	4 (36.4%)	2 (13.3%)	
MSSA	0 (0%)	5 (33.3%)	
*Enterococcus faecalis*	0 (0%)	1 (6.7%)	
*Streptococcus mutans*	0 (0%)	1 (6.7%)	
*Streptococcus oralis*	0 (0%)	1 (6.7%)	
*Corynebacterium jeikeium*	1 (9.1%)	1 (6.7%)	
*Clostridium paraputrificum*	1 (9.1%)	1 (6.7%)	
*Staphylococcus epidermidis*	0 (0%)	1 (6.7%)	
*Streptococcus parasanguinis*	1 (9.1%)	0 (0%)	
Bicuspid native valve	1 (9.1%)	4 (26.7%)	0.043
Previous AV block	1 (9.1%)	2 (13.3%)	0.738
Drug use history (negative)	11 (100%)	15 (100%)	0.738
Preoperative EF			0.498
Normal	6 (54.5%)	9 (60%)	
Borderline	3 (27.3%)	3 (20%)	
Moderately reduced	0 (0%)	2 (13.3%)	
Severely reduced	2 (18.2%)	1 (6.7%)	
30-day survival	10 (90.9%)	12 (80%)	0.446

AR=aortic root; ARA=aortic root abscess; AV=aortic valve; BI=BioIntegral; BMI=body mass index; BSA=body surface area; EF=ejection fraction; EUROSCORE II=European System for Cardiac Operative Risk Evaluation; FS=Freestyle™; IE=infective endocarditis; MSSA=methicillin-susceptible *Staphylococcus aureus*; PAOD=peripheral arterial occlusive disease; SCAAR=supracoronary ascending aorta replacement; STS=Society of Thoracic Surgeons

Before surgery, one (3.8%) patient suffered severe complications - critical limb ischemia and intestinal ischemia by septic cardioembolic closure of the common iliac artery and right common femoral artery.

Of the sample group (N=26), 26.9% underwent primary stand-alone Bentall surgery, while others had previously undergone an aortic surgery or a Bentall procedure after a previous AV replacement. Some patients also underwent other procedures such as mechanical Bentall, supracoronary ascending aorta replacement (SCAAR), David procedure with hemiarch replacement, and SCAAR for type-A aortic dissection. Four patients had AMC with pericardial plastic patch during their primary operation, and some required coronary artery bypass grafting or mitral valve repair. Among those who had redo surgeries within the sample group, five took place less than one year from their last cardiac procedure. The detailed data of the previous procedures is summarized in Table 1. Operative variables are summarized in Table 2. Ten patients received primary surgery. The mean period between the previous and the last cardiac surgery was 3.12 ± 4.87 years, after the previous surgery, while 4.33 ± 6.92 years for the BI and 2.13 ± 2.07 years for the FS prosthesis. Fifteen patients had a native AV, eight had an AV bioprosthesis, one had a mechanical prosthesis, and two had a mechanical conduit. Twelve (46.10%) patients were placed on CPB through peripheral cannulation before resternotomy. ARA was present in 15 (57.7%) patients. In five (19.23%) patients, an additional pericardial plastic patch of the AMC was applied due to extensive abscessed damage of the AR. Postoperative complications are summarized in Table 3. Most of the patients presented a combination of serious postoperative complications. The median ICU stay was four days, and the median duration of hospital stay was 29 days. The mortality rate during hospitalization was identical to the 30-day mortality rate, which stood at four out of 26 cases (15.40%). Two (7.70%) patients died intraoperatively due to irreversible AR damage. The most dominant postoperative complication was AV III block (N=9, 34.62%), while in four (15.4%) patients permanent pacemaker implantation was needed. The second most common was septic shock (N=4, 15.4%). Seven (26.93%) patients required new dialysis, and two patients (7.7%) suffered from transient neurologic dysfunction.

**Table 2:** Operative variables.

Parameters	BI (N=11)	FS (N=15)	*P*-value
Prosthesis size	24.45 ± 2.70	25.66 ± 1.79	0.181
Type of surgery			0.881
Current surgery type: primary			
Combined Bentall + AMC plastic patch	1	3	
Combined Bentall + CABG	3	1	
Combined Bentall + MVR	0 (0%)	1 (6.7%)	
Combined Bentall + MVRp + TVR + CABG	1 (9.1%)	1 (6.7%)	
Combined Bentall + AAR	0	1 (6.7%)	
Current surgery type: redo			
Bentall after previous AVR	4 (36.4%)	5 (33.3%)	
Redo-Bentall after previous Bentall + AAR	1 (9.1%)	1 (6.7%)	
Redo-Bentall after previous David + hemiarch replacement	1 (9.1%)	1 (6.7%)	
Redo-Bentall + CABG+ AMCR	1 (9.1%)	1 (6.7%)	
Re-redo-Bentall	0 (0%)	1 (6.7%)	
HLM cannulation site			0.391
Central	7 (63.6%)	7 (46.7%)	
Peripheral	4 (36.4%)	8 (53.3%)	
CPB time	162.82 ± 34.96	265.36 ± 90.66	0.002
Intraoperative death	1 (9.1%)	1 (6.7%)	0.89
Preoperative LV systolic function			0.299
Normal	6 (54.5%)	11 (73.3%)	
Moderately reduced	5 (45.5%)	3 (20%)	
Severely reduced	0 (0%)	1 (6.7%)	

AAR=ascending aorta replacement; AMC=aortomitral curtain; AMCR= aortomitral curtain reconstruction; AVR=aortic valve replacement; BI=BioIntegral; CABG=coronary artery bypass grafting; CPB=cardiopulmonary bypass; FS=Freestyle™; HLM=heart-lung machine; LV=left ventricular; MVR=mitral valve repair; MVRp=mitral valve replacement; TVR=tricuspid valve reconstruction

**Table 3:** Postoperative variables.

Parameters	BI (N=11)	FS (N=15)	*P*-value
Mechanical ventilation, hours	16.18 ± 15.24	33.26 ± 28.48	0.084
ICU stay, days	4.27 ± 3.85	9.71 ± 14.68	0.245
Postoperative in-hospital stay	33.09 ± 30.63	31.33 ± 24.25	0.872
Revision surgery during the first postoperative week			0.209
Not needed	11 (100%)	10 (66.7%)	
Yes, due to coronary artery- related complication	0 (0%)	1 (6.7%)	
Yes, due to pacemaker-related complication	0 (0%)	1 (6.7%)	
Yes, due to bleeding complication	0 (0%)	3 (20%)	
30-day postoperative valve function			0.337
Normal	9 (71.8%)	14 (93.3%)	
Abnormal	1 (9.1%)	0 (0%)	
No evaluation of possible cause of death	1 (9.1%)	1 (6.7%)	
Postop LV systolic function			0.629
Normal	7 (70%)	11 (73.3%)	
Moderately reduced	3 (30%)	3 (20%)	
Non evaluated due to intraoperative death	0 (0%)	1 (6.7%)	
In-hospital death			0.446
No	10 (90.9%)	12 (80%)	
Yes	1 (9.1%)	3 (20%)	
Cause of in-hospital death			0.012
Cardiac death, intraoperative irreversible aortic root damage	1 (9.1%)	1 (33.3%)	
Non-cardiac death, septic shock	0 (0%)	1 (33.3%)	
Non-cardiac death, intestinal ischemia	0 (0%)	1 (33.3%)	
Postoperative day of death	-	5.5 ± 2.12	

BI=BioIntegral; FS=Freestyle™; ICU=intensive care unit; LV=left ventricular

### Survival and Follow-up Results

All patients underwent surgery with ARR and bioconduit application. Of those, two died in the operation theater (7.7%) due to severe AR damage. Another two patients did not survive admission, so the total in-hospital mortality was four (15.38%). Thirty-day survival was 84.61% for all patients, 38.46% for BI, and 46.15% for FS. A comprehensive analysis of postoperative complications is presented in Table 4.

**Table 4:** Major postoperative complications.

Complications	BI (n=11)	FS (n=15)	0.733
None	1 (6.67)	2 (5.00)	
Septic shock	2 (13.33)	2 (5.00)	
Myocardial infarction	0 (0.00)	0 (0.00)	
Stroke	0 (0.00)	0 (0.00)	
Atrial fibrillation	1 (6.67)	2 (5.00)	
AV block III	4 (26.67)	1 (2.50)	
AV III with PPI	1 (6.67)	2 (5.00)	
Recurrent IE	0 (0.00)	0 (0.00)	
Cardiac arrest	1 (6.67)	0 (0.00)	
Acute heart failure	0 (0.00)	2 (5.00)	
Pericardial tamponade	1 (6.67)	0 (0.00)	
Delirium	0 (0.00)	3(7.50)	
Neutropenic sepsis	0 (0.00)	1 (2.50)	
Hemothorax	0 (0.00)	1 (2.50)	
Cardiogenic shock	0 (0.00)	1 (2.50)	
Cerebral hemorrhage	0 (0.00)	2 (5.00)	
Pulmonary bleeding	0 (0.00)	1 (2.50)	
RCA ostial narrowing	0 (0.00)	1 (2.50)	
Ventricular fibrillation	0 (0.00)	2 (5.00)	
Acute liver failure	0 (0.00)	1 (2.50)	
Non-relevant pericardial effusion	0 (0.00)	1 (2.50)	
Postoperative pacemaker malfunction	0 (0.00)	1 (2.50)	
Intracranial bleeding of an embolic lesion	0 (0.00)	1 (2.50)	
LSB	0 (0.00)	1 (2.50)	
VT	0 (0.00)	2 (5.00)	
Minimal pericardial effusion	0 (0.00)	1 (2.50)	
ROSC	1 (6.67)	1 (2.50)	
Pericardial effusion needing pericardiocentesis	1 (6.67)	1 (2.50)	
Same-day reoperation	1 (6.67)	1 (2.50)	
AKI	1 (6.67)	1 (2.50)	
Intraoperative death due to severely damaged heart basis rupture	1 (6.67)	1 (2.50)	
Candidemia sepsis	0 (0.00)	1 (2.50)	
Paravalvular abscess	0 (0.00)	1 (2.50)	
Abdominal compartment syndrome	0 (0.00)	1 (2.50)	
Mesenteric ischemia	0 (0.00)	1 (2.50)	
Total complications	16	40	0.733

AKI=acute kidney injury; AV=aortic valve; BI=BioIntegral; FS=Freestyle™; IE=infective endocarditis; LSB=left septal block; PPI=permanent pacemaker implantation; RCA=right coronary artery; ROSC=return of spontaneous circulation; VT=ventricular tachycardia

Follow-up was closed on December 31, 2023, and by then, ten (38.46%) patients were dead. Overall follow-up was 100% complete at a mean follow-up period of 4.33 ± 3.28 years (maximum follow-up was 128 months [10.67 years], and median follow-up was 4.25 years) postoperatively. Comprehensive details regarding the follow-up can be found in Table 5.

**Table 5:** Echocardiographic and postoperative follow-up.

Parameters	BI (N=11)	FS (N=15)	*P*-value
30-day LV EF, %	50.00 ± 8.82	55.09 ± 10.70	0.253
30-day TAPSE, mm	16.10 ± 2.28	15.90 ± 3.86	0.893
30-day PPG, mmHg	25.10 ± 14.63	30.54 ± 13.27	0.382
30-day MPG, mmHg	16.20 ± 7.90	15.45 ± 6.82	0.819
6-month LV EF, %	54.75 ± 3.45	53.10 ± 8.29	0.607
6-month TAPSE, mm	16.86 ± 2.54	15.90 ± 3.81	0.572
6-month PPG, mmHg	30.17 ± 15.95	58.60 ± 15.54	0.849
6-month MPG, mmHg	18.00 ± 9.08	14.90 ± 9.08	0.519
12-month LV EF, %	59.33 ± 4.62	50.75 ± 11.69	0.260
12-month TAPSE, mm	17.00 ± 2.64	15.12 ± 4.67	0.536
12-month PPG, mmHg	36.00 ± 13.11	23.37 ± 9.95	0.116
12-month MPG, mmHg	21.33 ± 7.50	14.50 ± 8.55	0.257
24-month LV EF, %	49.50 ± 13.43	47.25 ± 9.57	0.820
24-month TAPSE, mm	19.00 ± 5.65	15.00 ± 3.92	0.355
24-month PPG, mmHg	34.00 ± 18.38	22.50 ± 9.88	0.350
24-month MPG, mmHg	19.00 ± 8.48	12.75 ± 5.31	0.313
Survival in months	52.82 ± 45.26	51.56 ± 36.18	0.938

BI=BioIntegral; EF=ejection fraction; FS=Freestyle™; LV=left ventricular; MPG=mean pressure gradient; PPG=peak pressure gradient; TAPSE=tricuspid annular plane systolic excursion

Most common cause of death was non-cardiac. One (3.8%) patient was reoperated 10 months after admission in another hospital due to leakage and died one month later of cardiac cause.

Initial echocardiographic evaluation demonstrated favorable functional outcomes. Freedom from reoperation in follow-up was 80.76%. Four-year survival rate was 57.7% (N=15). Six BI patients survived > 4 years, and nine FS patients survived > 4 years.

### Correlations

**Fig. 1:**
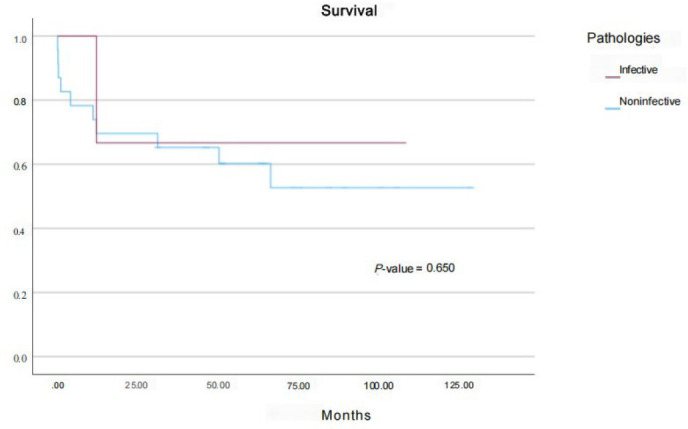
Survival per pathology type.

There was statistically significant dependence between previous aortic surgery and previous aortic dissection with SCAAR to the 30-day survival (chi-square [4.26] 12.254, *P*-value 0.016 and chi-square [1.26] 5.720, *P*-value 0.017, respectively). Eleven patients died in the follow-up of 17.04 ± 22.47 months, ten (90.9%) of them had received an ARR as redo surgery, showing a statistically significant dependence between previous cardiac surgery and follow-up death (chi-square [1.26] 6.949, *P*-value 0.008). There was no significant statistical difference between the survival time of the two prosthesis types, *P*-value was > 0.05 (0.889). We didn’t prove any significant difference (*P*-value > 0.005) regarding the survival between the infective and noninfective groups, as seen in Figure 1.

### Comparison Between the Groups

#### BioIntegral vs. Freestyle™ Bioconduit

**Fig. 2:**
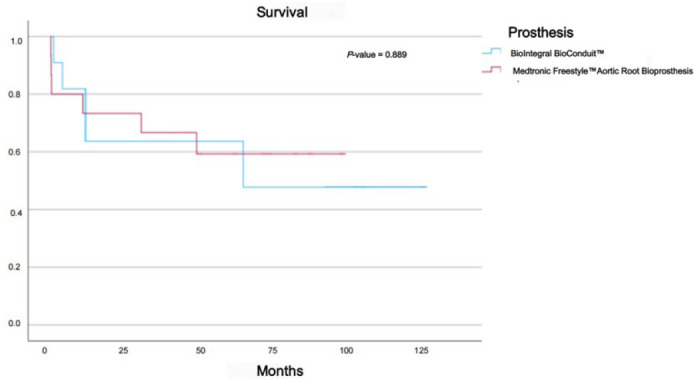
Survival per bioconduit type.

A comparison of different variables between the two conduits is to be seen in Tables 1-5. CPB time is shorter in BI *vs.* FS (162.82 ± 34.96 *vs.* 265.36 ± 90.66, *P*-value 0.002). As seen in Figures 1 and 2, there was no significant statistical survival difference (*P*-value 0.650). Mean survival for BI was 81 ± 37.81 months, and for FS it was 75.66 ± 19.65 months. There was no significant statistical difference regarding preoperative comorbidities or postoperative complications in both groups.

#### Aortic Root Abscess vs. Non-Aortic Root Abscess

Data are shown in Table 6. Patients with ARA presented at an older age than patients without ARA (67 ± 15.68 *vs.* 55.1 ± 17, *P* < 0.043) and received bigger prosthesis sizes (26.2 ± 1.68 *vs.* 24.33 ± 2.35, *P*=0.021). Echocardiographic, 12-month peak pressure gradient in the ARA group was higher than for non-ARA patients (35.5 ± 13.20 *vs.* 21.85 ± 8.05, *P*-value=0.029).

**Table 6:** Comparison of operative and follow-up variables in infective endocarditis cases with and without ARA.

Parameters	ARA N=15	NON-ARA N=10	*P*-value
Prosthesis size	24.33 ± 2.35	26.2 ± 1.68	0.021
Redo (year after last surgery)	4.7 ± 6.18	1.83 ± 2.5	0.106
Age	67 ± 15.68	55.1 ± 17.00	0.043
In-hospital stay, days	27.2 ± 20.77	41 ± 33.63	0.108
Intubation, hours	26.06 ± 24.87	27.4 ± 27.18	0.450
ICU stay, days	9.14 ± 14.19	5.3 ± 6.65	0.218
EuroSCORE II	18.16 ± 18.22	14.48 ± 20.66	0.322
STS score	2.68 ± 3.13	3.57 ± 5.96	0.316
Height, m	173.66 ± 8.34	175.4 ± 4.94	0.281
Weight, kg	76.86 ± 12.29	79.8 ± 8.43	0.259
BMI, kg/m^2^	25.52 ± 4.21	25.93 ± 3.00	0.398
BSA, m^2^	1.89 ± 0.18	1.95 ± 0.09	0.175
CPB time, minutes	228.26 ± 98.24	212.77 ± 74.52	0.344
Cross-clamping time, minutes	144.2 ± 55.14	136.1 ± 41.35	0.348
POD of death	2.2 ± 3.19	-	0.282
30-day LV EF, %	55.27 ± 7.37	52.11 ± 10.05	0.214
30-day TAPSE, mm	16.72 ± 2.72	15.33 ± 3.67	0.171
30-day PPG, mmHg	30.27 ± 14.19	26.66 ± 13.96	0.288
30-day MPG, mmHg	17.09 ± 7.82	15.22 ± 6.30	0.285
6-month LV EF, %	55.33 ± 6.18	52 ± 7.17	0.160
6-month TAPSE, mm	16.33 ± 3.46	16 ± 3.51	0.426
6-month PPG, mmHg	30.75 ± 10.23	28.71 ± 20.88	0.405
6-month MPG, mmHg	16.87 ± 4.76	16 ± 12.84	0.430
12-month LV EF, %	55.5 ± 5.00	51.71 ± 13.22	0.301
12-month TAPSE, mm	17 ± 3.36	14.85 ± 4.63	0.221
12-month PPG, mmHg	35.5 ± 13.20	21.85 ± 8.05	0.029
12-month MPG, mmHg	21.25 ± 10.87	13.57 ± 6.07	0.080
24-month LV EF, %	55.66 ± 4.93	40.33 ± 5.50	0.011
24-month TAPSE, mm	17 ± 2.00	15.66 ± 6.65	0.378
24-month PPG, mmHg	36 ± 9.84	16.66 ± 5.13	0.020
24-month MPG, mmHg	19.66 ± 5.03	10 ± 3.00	0.023
Postoperative survival in months	47.29 ± 43.01	58.1 ± 36.62	0.261

ARA=aortic root abscess; BMI=body mass index; BSA=body surface area; CPB=cardiopulmonary bypass; EF=ejection fraction; EUROSCORE II=European System for Cardiac Operative Risk Evaluation; ICU=intensive care unit; LV=left ventricular; MPG=mean pressure gradient; POD=postoperative day; PPG=peak pressure gradient; STS=Society of Thoracic Surgeons; TAPSE=tricuspid annular plane systolic excursion

## DISCUSSION

Surgical interventions on the AR have exhibited substantial advancements in recent years, thanks to the progress of surgical techniques and operative and postoperative approaches. However, due to the multiple severe diseases of the aortic bulb and the multi-morbid patients requiring ARR, ARR remains a challenge despite the rapid development in this field. In this study, we report our experience and results after urgent and emergent ARR with BI and FS bioconduits.

Esaki et al.^[20]^ have identified the risk factors associated with operative mortality related to ARR as including previous myocardial infarction, chronic obstructive pulmonary disease (COPD), and concurrent mitral valve surgery (MVS), while the long-term mortality increased with age and concomitant MVS. In our study, we confirmed these risk factors as well. Reoperative ARR is of significant potential high morbidity and mortality^[20]^. Interestingly, we showed a higher incidence of morbidity and mortality in reoperative cases, especially after previous surgery for aortic dissection. Additionally, in our study, one of the most dominant postoperative complications was the increased postoperative permanent pacemaker implantation rate, which as described in the literature in an independent long-term mortality risk factor^[21]^. Patel P.M. et al.^[1]^ reported an acceptable mid-term outcome of ARR with the FS conduit. In our study, despite the small number of patients, we could confirm this finding too.

Over time, the ARR with application of xenografts is gaining ground over the traditional ARR with mechanical conduits and is a proven exceptional solution in cases with different AR pathologies, including IE complicated with ARA and a destructed root. Bioroots offer comparable benefits to aortic allografts, as they enable the reconstruction of the AR following extensive debridement of infected tissues. Multiple reports indicate that bioroots exhibit remarkable durability, achieving a 94% rate of freedom from structural valve disease over a span of 14 years^[1^,^22^-^24]^. In our study, we observed favorable results in terms of clinical outcomes, with no notable occurrences of structural valve dysfunction or other significant conduit-related complications noted during the follow-up period. Interestingly, in our study, although the BI group had a higher operative risk of mortality, according to the Society of Thoracic Surgeons and the European System for Cardiac Operative Risk Evaluation II scores, we did not prove any significant statistical difference between the survival in the two bioconduit subgroups (*P*-value 0.889).

Based on the bibliography, operative mortality of ARR is reported with a range of 1% to 5% when performed in the setting of primary cardiac surgery^[25^-^28]^, and 2% to 18% in the case of reoperative AR surgery^[9^,^29]^. Esaki et al.^[20]^ showed a five-year survival after reoperative ARR of 74.0%, while previous ARR, prior to proximal aortic surgery, and concomitant arch replacement proved to be no risk factors for operative mortality. LeMaire et al.^[24]^ showed in a report of 132 patients with ARR using stentless xenografts, a survival of 85.6% ± 3.1% at one year and 77.8% ± 4.8% at five years. This approximates our findings, as we showed that the four-year survival was 57.7%. Comparing the two bioconduits, we proved significantly longer CPB times in the FS group, but no notable differences by terms of clinical outcome, survival, and graft functional performance as shown in Table 5. This is consistent with other studies showing no significant differences, but shorter CPB due to the simplified implantation technique of BI conduit^[22]^. Puehler et al.^[30]^ reported a 33-patient cohort who underwent Bentall procedure with BI conduit and showed 30-day mortality of 33%, mainly due to multiorgan failure, 3% graft reinfection with ARA, but a stable survival of patients after hospital discharge. We could not prove a significant difference in the survival correlated to the presence of ARA in the infective cases. This may be due to the small cohort volume.

### Limitations

The study has certain limitations due to its retrospective and non-randomized nature. Additionally, the selection of graft-conduit was based on the surgeon’s preference, experience, and surgery type. When comparing the two groups, significant disparities were observed in their risk profile and baseline characteristics since they were not entirely homogeneous. This study encompassed diverse clinical settings and patients who had previously undergone cardiac or aortic surgeries. This study includes urgent and emergency cases and should not be generalized in elective ARR cases.

## CONCLUSION

ARR presents a formidable surgical challenge due to the severity of its pathology. The choice between utilizing the BI or FS conduit does not appear to significantly impact ultimate outcomes in terms of operative mortality, survival, and follow-up valvular hemodynamics. Nonetheless, it is noteworthy that age, comorbidities, and concomitant surgery remain crucial factors that directly influence long-term survival and clinical outcomes. It should be emphasized that this study was conducted at a single center and thus its findings cannot be generalized or applied universally. The individualized assessment of patients requiring an ARR remains essential for achieving the best outcomes. Consequently, additional research with larger cohorts is warranted to attain a more comprehensive understanding of this complex entity.

## References

[1] Patel PM, Callahan M, Dong A, Wei J, Binongo J, Leshnower B (2022). Clinical outcomes using freestyle valve-valsalva graft composite
conduit for aortic root replacement. Ann Thorac Surg.

[2] Kouchoukos NT, Marshall WG (1986). Wedige-Stecher TA. Eleven-year experience with composite graft
replacement of the ascending aorta and aortic valve. J Thorac Cardiovasc Surg.

[3] Bentall H, De Bono A. (1968). A technique for complete replacement of the ascending
aorta. Thorax.

[4] Baumgartner H, Falk V, Bax JJ, De Bonis M, Hamm C, Holm PJ (2017). 2017 ESC/EACTS guidelines for the management of valvular heart
disease. Eur Heart J.

[5] Blehm A, Schurr P, Sorokin VA, Zianikal I, Kamiya H, Albert A (2014). Comparison of different surgical techniques in 112 consecutive
patients with aortic root operations: when should the valve be
spared?. J Heart Valve Dis.

[6] Lim E, Ali A, Theodorou P, Sousa I, Ashrafian H, Chamageorgakis T (2008). Longitudinal study of the profile and predictors of left
ventricular mass regression after stentless aortic valve
replacement. Ann Thorac Surg.

[7] Lehr EJ, Wang PZ, Oreopoulos A, Kanji H, Norris C, Macarthur R. (2011). Midterm outcomes and quality of life of aortic root replacement:
mechanical vs biological conduits. Can J Cardiol.

[8] Etz CD, Homann TM, Rane N, Bodian CA, Di Luozzo G, Plestis KA (2007). Aortic root reconstruction with a bioprosthetic valved conduit: a
consecutive series of 275 procedures. J Thorac Cardiovasc Surg.

[9] Kirsch EW, Radu NC, Mekontso-Dessap A, Hillion ML, Loisance D. (2006). Aortic root replacement after previous surgical intervention on
the aortic valve, aortic root, or ascending aorta. J Thorac Cardiovasc Surg.

[10] Paradis JM, Del Trigo M, Puri R, Rodés-Cabau J. (2015). Transcatheter valve-in-valve and valve-in-ring for treating
aortic and mitral surgical prosthetic dysfunction. J Am Coll Cardiol.

[11] Blehm A, Sorokin VA, Hartman M, Wai KL, Schmitz K, Lichtenberg A. (2015). Quality of life shift after aortic valve replacement in the era
of tavi: single-center class comparison study between different procedural
techniques. J Heart Valve Dis.

[12] De Paulis R, Scaffa R, Salica A, Weltert L, Chirichilli I. (2018). Biological solutions to aortic root replacement: valve-sparing
versus bioprosthetic conduit. J Vis Surg.

[13] Etz CD, Girrbach FF, von Aspern K, Battellini R, Dohmen P, Hoyer A (2013). Longevity after aortic root replacement: is the mechanically
valved conduit really the gold standard for
quinquagenarians?. Circulation.

[14] Galla JD, Lansman SL, Spielvogel D, Minanov OP, Ergin MA, Bodian CA (2002). Bioprosthetic valved conduit aortic root reconstruction: the
Mount Sinai experience. Ann Thorac Surg.

[15] Di Eusanio M, Murana G, Cefarelli M, Mazzola A, Di Bartolomeo R. (2014). The Bentall procedure with a biological valved conduit:
substitute options and techniques. Multimed Man Cardiothorac Surg.

[16] Doty DB, Cafferty A, Kon ND, Huysmans HA, Krause AH (1998). Westaby S. Medtronic freestyle aortic root bioprosthesis: implant
techniques. J Card Surg.

[17] Beckerman Z, Leshnower BG, McPherson L, Binongo JN, Lasanajak Y, Chen EP. (2018). The evidence in a bentall procedure with valsalva graft: is this
standard of care?. J Vis Surg.

[18] Abolhoda A, Yu S, Oyarzun JR, Allen KR, McCormick JR, Han S (1996). No-react detoxification process: a superior anticalcification
method for bioprostheses. Ann Thorac Surg.

[19] Di Eusanio M, Berretta P, Alfonsi J, Cefarelli M. (2019). Aortic root endocarditis: a biointegral bioconduit subannular
implantation. Ann Cardiothorac Surg.

[20] Esaki J, Leshnower BG, Binongo JN, Lasanajak Y, McPherson L, Thourani VH (2017). Reoperative aortic root replacement: outcome in a contemporary
series. J Thorac Cardiovasc Surg.

[21] Glaser N, Persson M, Dalén M, Sartipy U. (2021). Long-term outcomes associated with permanent pacemaker
implantation after surgical aortic valve replacement. JAMA Netw Open.

[22] Mehdiani A, Sorokin VA, Sule J, Smiris K, Stadnik D, Lichtenberg A (2020). Mid-term single-center outcomes of biointegral compared to
freestyle aortic conduit implantation. J Cardiovasc Surg (Torino).

[23] Isselbacher EM, Preventza O, Hamilton Black J, Augoustides JG, Beck AW, Writing Committee Members (2023). 2022 ACC/AHA guideline for the diagnosis and management of aortic
disease: a report of the American heart association/American college of
cardiology joint committee on clinical practice guidelines. J Thorac Cardiovasc Surg.

[24] LeMaire SA, Green SY, Sharma K, Cheung CK, Sameri A, Tsai PI (2009). Aortic root replacement with stentless porcine xenografts: early
and late outcomes in 132 patients. Ann Thorac Surg.

[25] Stamou SC, Williams ML, Gunn TM, Hagberg RC, Lobdell KW, Kouchoukos NT. (2015). Aortic root surgery in the United States: a report from the
society of thoracic surgeons database. J Thorac Cardiovasc Surg.

[26] Etz CD, Bischoff MS, Bodian C, Roder F, Brenner R, Griepp RB (2010). The Bentall procedure: is it the gold standard? A series of 597
consecutive cases. J Thorac Cardiovasc Surg.

[27] Zehr KJ, Orszulak TA, Mullany CJ, Matloobi A, Daly RC, Dearani JA (2004). Surgery for aneurysms of the aortic root: a 30-year
experience. Circulation.

[28] Gott VL, Greene PS, Alejo DE, Cameron DE, Naftel DC, Miller DC (1999). Replacement of the aortic root in patients with Marfan's
syndrome. N Engl J Med.

[29] Malvindi PG, van Putte BP, Heijmen RH, Schepens MA, Morshuis WJ. (2010). Reoperations on the aortic root: experience in 46
patients. Ann Thorac Surg.

[30] Puehler T, Freitag-Wolf S, Friedrich C, Salem M, Renner J, Cremer J (2020). Outcomes of patients after implantation of the pericardial
all-biological valve no-react aortic conduit (biointegral) for root
replacement in complex surgical procedures. Thorac Cardiovasc Surg.

